# The Effect of Secondary Metabolites Produced by *Serratia marcescens* on *Aedes aegypti* and Its Microbiota

**DOI:** 10.3389/fmicb.2021.645701

**Published:** 2021-07-07

**Authors:** Katy Heu, Ottavia Romoli, Johan Claes Schönbeck, Rachel Ajenoe, Yanouk Epelboin, Verena Kircher, Emeline Houël, Yannick Estevez, Mathilde Gendrin

**Affiliations:** ^1^Microbiota of Insect Vectors Group, Institut Pasteur de la Guyane, Cayenne, France; ^2^CNRS, UMR EcoFoG, AgroParisTech, Cirad, INRAE, Université des Antilles, Université de Guyane, Cayenne, France; ^3^Parasites and Insect Vectors Department, Institut Pasteur, Paris, France

**Keywords:** *Serratia*, *Aedes aegypti*, secondary metabolite, prodigiosin, microbiota, serratamolide, serrawettin, Hfq

## Abstract

*Serratia marcescens* is a bacterial species widely found in the environment, which very efficiently colonizes mosquitoes. In this study, we isolated a red-pigmented *S. marcescens* strain from our mosquito colony (called *S. marcescens* VA). This red pigmentation is caused by the production of prodigiosin, a molecule with antibacterial properties. To investigate the role of prodigiosin on mosquito-*S. marcescens* interactions, we produced two white mutants of *S. marcescens* VA by random mutagenesis. Whole genome sequencing and chemical analyses suggest that one mutant has a nonsense mutation in the gene encoding prodigiosin synthase, while the other one is deficient in the production of several types of secondary metabolites including prodigiosin and serratamolide. We used our mutants to investigate how *S. marcescens* secondary metabolites affect the mosquito and its microbiota. Our *in vitro* tests indicated that *S. marcescens* VA inhibits the growth of several mosquito microbiota isolates using a combination of prodigiosin and other secondary metabolites, corroborating published data. This strain requires secondary metabolites other than prodigiosin for its proteolytic and hemolytic activities. In the mosquito, we observed that *S. marcescens* VA is highly virulent to larvae in a prodigiosin-dependent manner, while its virulence on adults is lower and largely depends on other metabolites.

## Introduction

*Aedes aegypti* mosquitoes are the main vectors of several arthropod-borne viruses of importance to human health, including dengue, Zika, and chikungunya viruses. The ability of mosquitoes to transmit diseases is influenced by several environmental and intrinsic factors, which affect mosquito population size, lifespan, and interactions with viruses. These factors notably include rainfall, temperature, mosquito antiviral immunity, as well as the microbial communities harbored by the mosquito epithelia (Lefèvre et al., 2013; Lee et al., 2019; Scolari et al., 2019). Interestingly, some studies have found that antibiotic treatment of *Ae. aegypti* increased permissiveness of mosquitoes to arboviral development (vector competence), while others found that it did not affect viral infection, suggesting that this impact may depend on initial microbiota composition (Xi et al., 2008; Audsley et al., 2017).

Comparative studies of the influence of several bacterial strains further showed strain-specific impacts on vector competence, where some bacteria were found to protect mosquitoes against virus infection while others have no, or even a positive effect on arboviral infection (Ramirez et al., 2012; Wu et al., 2019). Increase in vector competence has notably been found in the case of several *Serratia* species, notably *Serratia marcescens* (Apte-Deshpande et al., 2012; Wu et al., 2019). This is due to the secretion of *SmEnhancin*, an enzyme which specifically degrades mucins in the gut of *Ae. aegypti* (but not in other mosquitoes) and limits the natural protection due to the gut mucus (Wu et al., 2019). This bacterial species, which has a very strong colonization ability in mosquitoes (Wang et al., 2017), is however also regarded with high interest as a potential malaria transmission blocker (Pike et al., 2017). Indeed, genetic manipulation of a *S. marcescens* strain to artificially produce arthropod antimicrobial peptides strongly reduces infection of *Anopheles* mosquitoes by malaria parasites, hence, may be used to block transmission *via* paratransgenesis (manipulation of symbionts to modify the host’s phenotype). Some *S. marcescens* strains produce a red pigment called prodigiosin, which has some larvicidal and pupicidal activity in *Ae. aegypti* (Patil et al., 2011; Suryawanshi et al., 2015), yet its impact on mosquito physiology has not been investigated in detail. Moreover, prodigiosin has antimicrobial and antifungal properties (Williamson et al., 2006) which might impact other members of the mosquito microbiota. In this study, we characterized a prodigiosin-producing strain of *S. marcescens* which colonized our mosquito colony. We investigated whether secondary metabolite production by this bacterial strain affects microbe-microbe interactions and *Serratia* virulence in the mosquito.

## Results

### Isolation of the *S. marcescens* VA Strain and Production of Prodigiosin-Deficient Mutants

When rearing our colony of *Ae. aegypti*, we observed a pink staining of the (non-autoclaved) sugar solution used to feed mosquitoes without any obvious loss in the colony. Concomitantly, we also observed that after rearing field-collected *Anopheles darlingi* (also called *Nyssorhynchus darlingi*) for one single generation in the same insectary using the same sugar source, 8/11 gut homogenates gave rise to red colonies after overnight incubation on LB (lysogeny broth) agar at 25°C. A chosen isolate of these colonies was identified as *S. marcescens via* biochemical analyses and sequencing of *16S* ribosomal DNA. We also noticed some mortality at the larval and pupal stage among *An. darlingi* mosquitoes, where dying individuals appeared pink and red colonies could be grown from their homogenates. As the pink sugar solution had been regularly observed in our insectary in the past and is not observed since its autoclaving has become a standard procedure, we hypothesize that the sugar meal may have been the source of this bacterial contamination in our colony. We called this strain *S. marcescens* VA, in reference to our entomology facility (Vectopole Amazonien). From this stage onward, all the experimental work in mosquitoes was performed on *Ae. aegypti*, as we did not have enough *An. darlingi* for further characterization.

**FIGURE 1:**
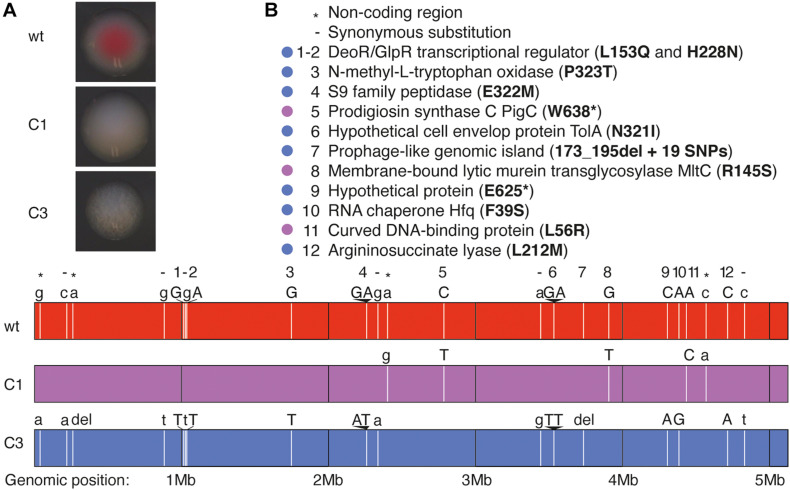
Whole-genome sequencing analysis of *S. marcescens* VA prodigiosin-deficient mutants. **(A)** Pictures showing the aspect of colonies of wt *S. marcescens* VA and of its C1 and C3 mutants. **(B)** Description of the mutated loci in C1 and C3 mutants. *Lower panel*: Each vertical white bar represents the locus of a mutation, and the original (wt) and mutated (C1 or C3) nucleotides are indicated above the corresponding colored bars representing each genome. Uppercase letters indicate non-synonymous substitutions, while lower case letters and - indicate synonymous substitutions. The symbols * indicate mutations in non-coding regions. *Upper panel*: the legend indicates the gene where each mutation is located and the corresponding mutation in the amino acid chain. The color-code indicates the bacterial strains in which the mutations have been identified (purple: C1, blue: C3).

*Serratia marcescens* owes its red color to prodigiosin, a bacteriostatic pigment with larvicidal activity in mosquitoes, which is produced at 30°C but not at 37°C. As the average temperature in French Guiana is close to 30°C, we hypothesized that prodigiosin may impact mosquito physiology, both directly and *via* bacteria-bacteria interactions in the mosquito gut. To test this, we generated prodigiosin-deficient mutants by random mutagenesis *via* UV treatment of a *S. marcescens* VA culture and isolation of white clones. Among the white colonies, we selected two clones, C1 and C3, which stay white even after 4 days of culture at 30°C and for at least two passages. We observed that colonies of both mutants had a slightly different morphology, C3 colonies having a granular appearance while C1 colonies looked more homogeneous (Figure 1A). Growth kinetics were similar in wild-type (wt) and mutant strains (Supplementary Figure 1).

### Genome Sequencing of wt and Mutant *S. marcescens* VA

We sequenced the genomes of our three clones using MiSeq technology. We identified only five single-nucleotide polymorphisms (SNPs) in the C1 mutant, including two in non-coding regions and three non-synonymous substitutions in coding regions (Figure 1B). Two of these mutations resulted in amino acid substitutions while the third introduced a stop codon in the *pigC* gene, which encodes prodigiosin synthase, the last enzyme of prodigiosin biosynthesis. We therefore hypothesize that the white color is linked to the lack of prodigiosin synthase.

The genome of mutant C3 contains more mutations (Figure 1B): 1 SNP in non-coding regions, one single nucleotide deletion in a non-coding region, six synonymous substitutions and 10 non-synonymous substitutions (at eight locations) in coding regions, and a deletion followed with 19 SNPs in a prophage like-island. As the alignment was poorer at this specific locus, we cannot precisely determine the size of the deletion. We did not detect any mutation in genes encoding enzymes of the prodigiosin synthesis pathway, yet we found a mutation in the gene encoding RNA chaperone Hfq, known to control the production of several secondary metabolites including prodigiosin (Wilf et al., 2011).

### Effect of *S. marcescens* VA on the Mosquito Microbiota

**FIGURE 2:**
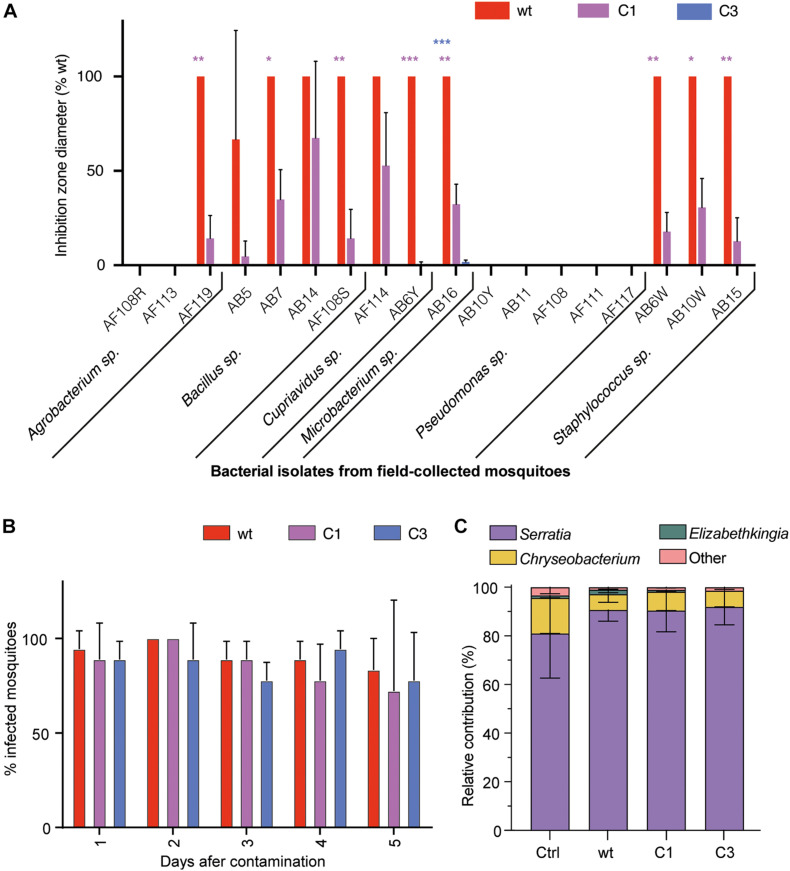
Interaction of *S. marcescens* VA with the mosquito microbiota. **(A)** Inhibition zone diameter of the three strains on 18 bacterial strains isolated from the gut of field-collected mosquitoes. **(B)** Colonization success showed as the proportion of infected mosquitoes 1–5 days after feeding with a contaminated sugar solution (OD_600 nm_ = 1). **(C)** Relative contribution of the main bacterial genera found by high-throughput sequencing of 16S in mosquito guts 24 h after oral infection. Data show the average ± SEM of three independent replicates. *p < 0.05; **p < 0.01; ***p < 0.001.

We first tested the impact of *S. marcescens* VA wt, CA and C3 on the mosquito microbiota. We quantified the antibacterial activity of *S. marcescens* VA and its white mutants by measuring their inhibition diameters on LB agar against several bacteria isolated from field-collected mosquitoes. *S. marcescens* VA inhibited the growth of 11/18 tested isolates belonging to the *Agrobacterium*, *Bacillus*, *Cupriavidus*, *Microbacterium*, and *Staphylococcus* genera (Figure 2A). The inhibition diameter of the mutant C1 was significantly reduced for eight of these 11 isolates, which belong to all of these five genera (p_adj_ < 0.05, *t*-test with Bonferroni–Dunn correction). We did not detect any growth inhibition by C3 except a minor inhibitory effect on the isolate of *Microbacterium* sp. (the inhibition zone was 98% smaller than that of wt, p_adj_ < 0.001). We then tested whether *S. marcescens* VA negatively impacts other bacteria within the mosquito gut, and thus affects microbiota composition. To this aim, we infected mosquitoes with bacteria by feeding them with a contaminated sugar solution [optical density (OD)_600 nm_ = 1; estimated 10^8^ CFU/mL]. We took advantage of the resistance of our strain to a penicillin-streptomycin cocktail to test the colonization success, which was high in wt and in mutants (Figure 2B; two-way ANOVA, day: *p* = 0.30; bacterial strain: *p* = 0.63; interaction: *p* = 0.95). We sequenced the V3–V4 region of the *16S* rRNA bacterial gene from pools of dissected midguts of our *Ae. aegypti* colony, 3 days after oral infection. However, we found that our colony was already dominated by penicillin-streptomycin susceptible *Serratia* sp., hence, this experiment did not allow us to conclude whether *S. marcescens* VA affects microbiota composition (Figure 2C). We also did not detect any impact of *S. marcescens* VA colonization on the microbiota alpha-diversity (Supplementary Figure 2). Together, our data indicate that *S. marcescens* VA is very efficient at colonizing mosquitoes at the tested concentration regardless of prodigiosin synthesis, and that the prodigiosin-producing wt strain is bacteriostatic on most of the tested bacterial isolates from field-collected mosquitoes.

### Enzymatic Activity of *S. marcescens* VA Secreted Factors

**FIGURE 3:**
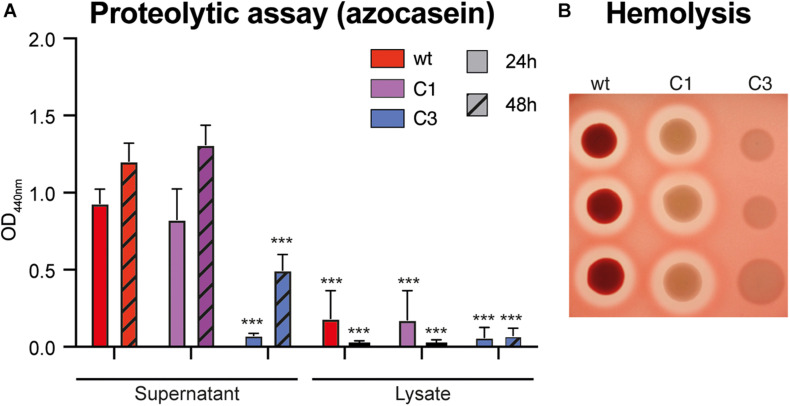
Proteolytic and hemolytic activity of *S. marcescens* VA. **(A)** Proteolytic activity of wt, C1 and C3 *S. marcescens* VA extracts. Tests were performed using an azocasein colorimetric assay on extracts coming from cultured incubation for 24 or 48 h. Data show the average ± SEM of three independent replicates. ***p < 0.001. **(B)** Hemolytic activity on blood-agar containing human red-blood cells. The picture shows the result of one representative experiment with three technical replicates for each strain. Three independent replicates were performed.

Bacteria colonizing mosquitoes have been reported to have some proteolytic and/or hemolytic activity, which affect mosquito digestion dynamics and vector competence (de Gaio et al., 2011; Wu et al., 2019; Jupatanakul et al., 2020). Using an azocasein-based colorimetric assay, we observed that *S. marcescens* VA secretes a proteolytic factor in the supernatant during the first 24 h of the culture and that this factor is also produced in C1 mutant but not in C3 (Figure 3A; C1-wt – −12%, p_adj_ > 0.99; C3-wt – −92%, p_adj_ < 0.0001; two-way ANOVA + Bonferroni’s multiple comparison tests). When testing cultures after 48 h, we observed that C3 did produce some proteolytic factor, but at a lower rate (Figure 3A; C1-wt – +10%, p_adj_ > 0.99; wt vs C3 – −58%, p_adj_ < 0.0001). Proteolytic activity in the bacterial lysate was significantly lower than in the supernatant at both time points (Figure 3A; all comparisons vs wt_*supernatant–*24 h_: p_adj_ < 0.0001). Considering hemolytic activity, we found that wt induces some hemolysis on blood agar, which was also present in C1 and lost in C3 (Figure 3B). This hemolytic activity was lost when wt bacteria were cultured at 37°C. However, when quantifying hemolysis in liquid culture, we did not find any difference between wt and either mutant; all of the strains had a similar hemolytic activity to our SDS (sodium dodecyl sulfate)-based positive control (Supplementary Figure 3). Hence, our data indicate that *S. marcescens* VA exhibits a prodigiosin-independent proteolytic and hemolytic activity.

### Characterization of Prodiginins and Serratamolides *via* HPLC and NMR Analyses

**FIGURE 4:**
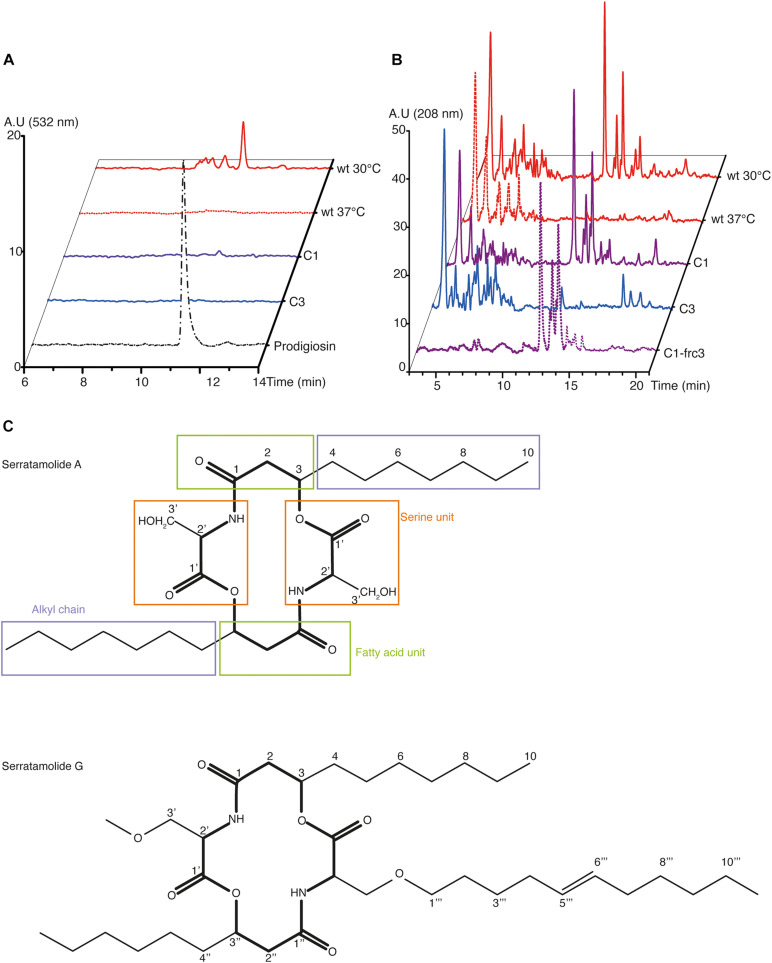
HPLC and NMR analyses of the secondary metabolites produced by *S. marcescens* VA. **(A,B)** HPLC profile of *S. marcescens* VA extracts at the wavelengths reported to detect prodigiosin (532 nm, **A**) and serratamolide (208 nm, **B**). In **(A)**, the profile of purified prodigiosin was used as a control. In **(B)**, C1-frc3 shows data from the methanolic fraction, which has been analyzed *via* NMR. Data are representative of two independent experiments, and of one for C1-frc3. A. U, arbitrary units. **(C)** Structure of serratamolide A and G molecules, where the core indicated in bold. Serratamolide A encompasses a central symmetry and its different parts described in Table 1 are indicated in colored boxes: fatty acid unit in green, alkyl chain in purple, and serine unit in orange.

In line with our genome sequencing data, we hypothesized that the observed differences between both mutants may be linked to an additional deficiency of C3 in the production of serratamolide (also called serrawettin), a hemolytic secondary metabolite that is regulated in a temperature-dependent manner downstream the same pathway as prodigiosin under the control of the chaperone Hfq (Tanikawa et al., 2006; Shanks et al., 2012). To test this, we performed an acidified-ethanol extraction after culturing our three strains at 30°C and the wt strain at 37°C, as a negative control, and we qualified their metabolites by high-performance liquid chromatography (HPLC). As expected, we observed that the peak matching with purified prodigiosin, detected at 532 nm, was observed in extracts from *S. marcescens* VA cultured at 30°C, but neither after culture at 37°C nor in both mutants (Figure 4A). We observed several peaks around that of purified prodigiosin, which is consistent with a previous report indicating that seven different prodigiosin-related compounds (prodiginins) can be synthesized by *S. marcescens* (Eckelmann et al., 2018).

Considering serratamolide, we had no purified compound to formally identify its peak, but it is known to be detectable at 208 nm (Dwivedi et al., 2008). At this wavelength, we observed a family of peaks which is found in the wt at 30°C and not at 37°C, which makes them suitable candidates for serratamolide (Figure 4B). This family of peaks was not detected at 254, 280, and 532 nm (Figure 4A and Supplementary Figure 4). Such a family of multiple peaks is consistent with the fact that 26 different serratamolide metabolites have been reported to be produced by *S. marcescens* (Eckelmann et al., 2018). These peaks were also present in C1 extracts, but absent from C3 extracts (Figure 4B). To analyze whether they correspond to serratamolides, we purified these fractions from the C1 extract. We checked that the selected fractions showed a similar family of products, with an absorption maximum of 208 nm using HPLC analysis (Figures 4A,B and Supplementary Figure 4). We then submitted this fraction to nuclear magnetic resonance (NMR) analysis. NMR data indicated the presence of the characteristic serine and fatty acid moieties of serratamolides (Figure 4C and Table 1; Dwivedi et al., 2008). The two carbonyl signals were inferred from Heteronuclear Multiple Bond Correlation analysis (HMBC), at 171.8 and 171.0 ppm, and correspond to C1 and C′1, respectively. ^1^H signals and 2D correlations are consistent with the presence of a C_10_ alkyl chain attached to the serine unit through its hydroxy group and we clearly identified the alkyl chain terminal methyl group (δ_H_ 0.90 ppm; t; *J* = 7.0 Hz; δ_*C*_ 14.2 ppm). These structural elements indicate the presence of serratamolide A or very similar compounds (Soto-Cerrato et al., 2005; Dwivedi et al., 2008; Zhu et al., 2018). Finally, we detected additional signals that are characteristic of an unsaturated branch unit (notably δ_H_ 5.35 ppm; t; *J* = 4.7 Hz; δ_*C*_, 130.6 ppm, alongside with δ_*C*_ 136.3 ppm inferred from HMBC analysis, and COSY correlations between signals at δ_H_ 5.35, 2.04, and 1.31 ppm). A methoxy group (δ_H_ 3.65 ppm; s; δ_*C*_ 51.7 ppm) may also be part of the serratamolide structures, similar to previous reports on serratamolide G (Zhu et al., 2018) and overall consistent with the great structural diversity of this family of compounds (Eckelmann et al., 2018). Together, our data indicate that *S. marcescens* VA produces prodigiosin and serratamolides, probably dominated with serratamolide A. They further show that C1 produces serratamolides but no prodigiosin and that C3 is impaired in the production of both types of compounds.

**TABLE 1:** NMR data (CD_3_OD) obtained from fraction 3 spectrum and corresponding to serratamolides.

Position	^1^H: δ (ppm); multiplicity; *J* (Hz)	^13^C: δ (ppm) according to HSQC and HMBC	HMBC (^1^H → ^13^C)	COSY (^1^H → ^1^H)
***Serine unit***				

1′		171.0; C		
2′	4.48; dd; *J* = 3.5; 3.1 Hz	56.0; CH		3′a; 3′b
3′	3′a: 4.08; dd; *J* = 10.6; 3.5 Hz, 3′b: 3.83; dd; *J* = 10.9; 3.1 Hz	62.9; CH_2_		2′; 3′b
			C′1	2′; 3′a

***Fatty acid unit***				

1		171.8; C		
2	2a: 2.68; dd; *J* = 13.7; 5.1 Hz, 2b: 2.34; dd; *J* = 13.3; 2.4 Hz	40.9; CH_2_	C1; C3	2a; 3
			C1	2b; 3
3	5.30; m	72.9; CH		4; 2a; 2b

***C_10_ alkyl chain***				

4	1.67; m	33.5; CH_2_		3, -(CH2)n-
-(CH2)n-	1.29; m	23.6; 26.8; 30.2; 30.4; 32.8		-CH3
-CH3	0.90; t; *J* = 7.0 Hz	14.2; CH_3_		-(CH2)n-

-OCH_3_	3.65; s	51.7; CH_3_		

-NH	7.90; d; *J* = 8.2 Hz			

*Chemical shifts (δ) are in ppm downfield from tetramethylsilane (TMS), and coupling constants (*J*) are in Hz. s, singlet; d, doublet; t, triplet; m, multiplet; HSQC, Heteronuclear Single Quantum Coherence; HMBC, Heteronuclear Multiple Bond Correlation.*

### Effect of *S. marcescens* VA on Mosquito Fitness

**FIGURE 5:**
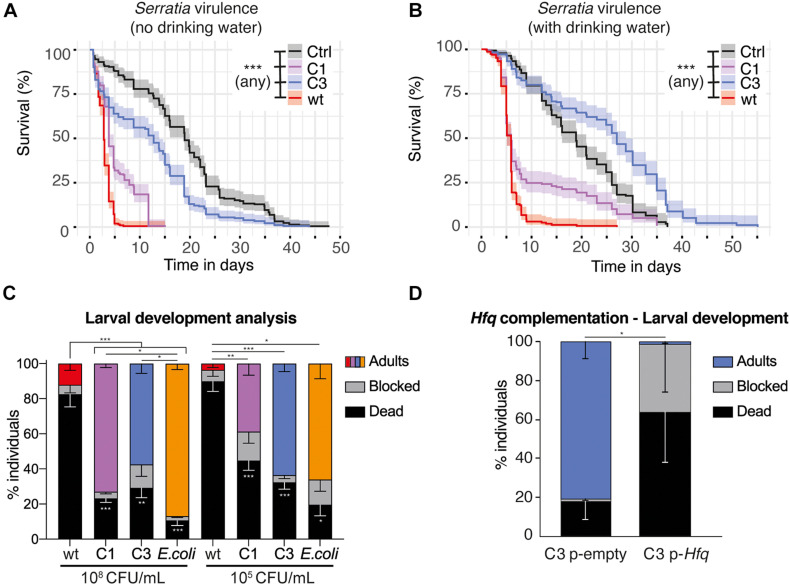
Impact of *S. marcescens* VA on adult and larval *Ae. aegypti*. **(A,B)** Survival of adult females fed with a sugar solution contaminated with a concentrated suspension of *S. marcescens* (OD_600 nm_ = 80). In **(B)**, mosquitoes were provided with drinking water in addition to the infectious meal. **(C,D)**, Development success to adulthood of larvae contaminated with *S. marcescens* from the first larval instar. In **(C)**, the concentration of the bacterial suspension at the beginning of the experiment is indicated below. CFU, colony forming unit. In **(D)**, larvae are provided with C3 carrying an empty pBBR1MCS-2 plasmid or an *Hfq*-containing pBBR1MCS-2, at a 10^8^ CFU/mL concentration. Data show the results of 2 **(A,B)**, 7 **(C)**, and 3 **(D)** independent replicates. Confidence intervals are shown in **(A,B)** and SEM in **(C,D)**. *p < 0.05; **p < 0.01; ***p < 0.001.

We performed further characterization of the virulence of *S. marcescens* VA in *Ae. aegypti*. A low infection dose (as used in Figures 2B,C; OD_600 nm_ = 1) caused a slight, yet significant reduction in survival, which was not affected in any mutant (Supplementary Figure 5A). We did not detect any significant difference between any strain on egg laying, hatching rate and sex ratio of the progeny (Supplementary Figures 5B,C). We then infected *Ae. aegypti* by providing a sugar solution mixed with a concentrated bacterial suspension (80-fold concentrated suspension compared to OD_600 nm_ = 1, referred to here as OD_600 nm_ = 80). *S. marcescens* VA wt infection was highly lethal to mosquitoes, causing 65 and 85% mortality by day 3 and day 4 post-infection respectively. C1 showed a slight reduction in virulence whereas C3 mutants were much less virulent (Figure 5A). We observed that infected mosquitoes survived longer when they had access to water in addition to the infectious bacteria (Figure 5B). This extension in lifespan was particularly marked with the case of C3 colonization, which even appeared to extend the lifespan of mosquitoes compared to controls provided a sugar solution and water.

Previous studies reported that purified prodigiosin caused some mortality to *Ae. aegypti* larvae, and that *S. marcescens* also secretes proteases and chitinases which have a larvicidal activity (Jupatanakul et al., 2020). On the other side, bacteria are required for normal larval development, and we have set up methods to test whether specific bacteria are able to support larval development (Coon et al., 2014; Romoli and Gendrin, 2020). Using our protocol, 100% sterile larvae are blocked at the first instar unless bacteria are added in the medium. We tested whether larval mono-colonization with *S. marcescens* VA was able to support development and/or whether it was killing larvae. When using our conventional concentration of the bacterial suspension (10^8^ CFU/mL), we found that only C1 and C3 efficiently support development, albeit to a lesser extent compared to the *Escherichia coli* positive control (Figure 5C, *p* < 0.001, mixed effect model; wt vs all: p_adj_ < 0.001; C1 vs *E. coli*: p_adj_ = 0.02; C3 vs *E. coli*: p_adj_ = 0.01; C1 vs C3: p_adj_ = 0.08, ns, Tukey’s multiple comparisons test). *S. marcescens* VA wt supports the development of 12% of the larvae, but also killed over 80% of the individuals. This suggests that *S. marcescens* is metabolically able to support development, but also has a strong larvicidal activity, most likely due to prodiginins as it is strongly reduced in C1. We then tested whether this impact on development was different with a lower bacterial concentration (10^5^ CFU/mL, Figure 5C). Again, wt was highly virulent, killing 90% of the larvae. We observed that 63% of the larvae develop to adulthood with C3, similar to the *E. coli* positive control, and detected a slightly lower success with C1 (39%; *p* < 0.001, mixed effect model; wt vs C1: p_adj_ = 0.01; wt vs C3: p_adj_ < 0.001; wt vs *E. coli*: p_adj_ = 0.02; C1 vs C3: p_adj_ = 0.07; C1 vs *E. coli*: p_adj_ = 0.28; C3 vs *E. coli*: p_adj_ = 1, Tukey’s multiple comparisons test). Intriguingly, development success was reproducibly higher when providing the high dose of C1 than the lower one, while this was not observed with C3 (C1 10^8^ vs C1 10^5^: p_adj_ = 0.03; C3 10^8^ vs C3 10^5^: p_adj_ = 0.80, Tukey’s multiple comparisons test). Together, we found that *S. marcescens* VA has only a minor impact on mosquito fitness when adults are infected at an intermediate dose (OD_600 nm_ = 1), while it is virulent in adults at a higher dose and in larvae at both concentrations tested (Figures 5A–C and Supplementary Figure 5). Our results also suggest that the prodiginins strongly participate in the virulence toward larvae, but have a minor impact in adults, where other secondary metabolites contribute to virulence.

To investigate potential larvicidal effects of *S. marcescens* VA metabolites in the absence of the bacterium itself, we tested whether a crude bacterial extract caused any lethality in third-to-fourth-instar larvae. Again, we used acidified-ethanol extracts from each strain after culture at 30°C, using as a negative control a wt crude extract after culture at 37°C, and quantified the effect of these extracts on third-to-fourth-instar larvae. As shown in Supplementary Figure 6, the crude extract of *S. marcescens* VA caused some significant lethality in larvae with an average LC_50_ (lethal concentration of 50% of the population) of 480 and 323 ppm if extracts were prepared 24 and 48 h after culture inoculation, respectively (wt 30°C vs wt 37°C, 24 h – *p* = 0.023, 48 h – *p* = 0.047, ANOVA with Bonferroni correction). When extracting from 24 h-old cultures, we found that C1 had a significant larvicidal activity, while C3 did not show any larvicidal activity compared to the negative control (C1 vs wt 37°C, *p* = 0.029; C3 vs wt 37°C, *p* > 0.99). Using extracts from 48 h-old cultures, data indicate some larvicidal activity of both C1 and C3, yet differences with the negative control are not significant (C1 vs wt 37°C, *p* = 0.17; C3 vs wt 37°C, *p* = 0.22). We also noted that the larvicidal activity was lost when extracts were kept, even frozen, for several weeks, indicating that the larvicidal compounds that we extracted are not sufficiently stable for any mosquitocidal application. Thus, in this setup we did not observe any impact of prodigiosin itself, but rather of other secondary metabolites produced under a temperature-dependent control.

### *Hfq*-Complementation Restores C3 Virulence

Our sequencing data indicates that C3 carries several mutations. To investigate which mutation is responsible for the observed phenotypes in C3, we tested whether the complementation of the genes encompassing these mutations resulted in red colonies. We amplified the wt sequences of the seven corresponding genes (Figure 1B) and cloned them into the expression vector pBBR1MSC-2 in C3. The process was successful six genes, i.e., all except N-methyl-L-tryptophan oxidase, and among them the only complementation resulting in red colonies was with *Hfq* (Supplementary Figure 7). We thus focused on *Hfq* for further characterization of the impact of complementation. Indeed, these results were not surprising, as *Hfq* encodes a chaperone controlling the production of several secondary metabolites including prodigiosin and serratamolide (Wilf et al., 2011). When comparing the impact of *Hfq*-complemented C3 with C3 carrying an empty plasmid, we observed that Hfq restores the virulence of *S. marcescens* VA during larval development, significantly decreasing the percentage of fully developed adults (Figure 5D, mixed effect model: % adults, *p* = 0.011; % dead, *p* = 0.16; % blocked, *p* = 0.25).

## Discussion

*Serratia marcescens* is a bacterium that efficiently colonizes mosquitoes and its interactions with several species of mosquitoes have therefore received much interest in the recent years. In this study, we characterized a prodigiosin-producing strain, which efficiently colonizes *Anopheles* and *Aedes* mosquitoes and is virulent in mosquitoes, particularly at the larval stage.

We used a random mutagenesis approach and selected two strains that did not produce prodigiosin for phenotypic characterization. Among them, C1 was found to only carry three non-synonymous mutations including a single nonsense mutation. The latter affects prodigiosin-synthase, hence phenotypes observed in C1 are likely consequences of the lack of prodigiosin. These phenotypes include firstly a reduced antimicrobial activity against eight bacterial strains belonging to four different classes (Alphaproteobacteria, Betaproteobacteria, Bacilli, and Actinobacteria) and secondly, an attenuated virulence in mosquitoes, particularly at the larval stage. This is consistent with a previous report of a larvicidal and pupicidal effect of purified prodigiosin in *Ae. aegypti* (Suryawanshi et al., 2015).

The genome of C3 includes a larger amount of mutations, including the RNA chaperone Hfq, which is involved in the regulation of the production of secondary metabolites including prodigiosin, serratamolide, and a carbapenem antibiotic (Wilf et al., 2011). We observed that C3 is highly affected in antimicrobial activity and has impaired production of prodigiosin and serratamolides. These phenotypes are consistent with a deficient regulation of secondary-metabolite production downstream to Hfq. We validated that the complementation of *Hfq* in C3 restored the bacterial pigmentation and larvicidal effect. The loss of these other metabolites affected proteolysis and hemolysis activity of *S. marcescens* VA, its antimicrobial activity as well as its virulence in adults. Whilst we did not identify the specific metabolites responsible for such phenotypes, serratamolide is a known hemolytic factor, previously found to contribute *in vitro* to the virulence of *S. marcescens* by increasing its resistance against phagocytosis by human polymorphonuclear leukocytes (Miyazaki et al., 1993; Shanks et al., 2012). Contrary to adults, bacterial virulence toward larvae seeme to rely on prodiginins, but not on the other secondary metabolites, as we did not detect any significant difference between both mutants in larval development success. When using 10^8^ CFU/mL, we detect some residual virulence of C1 and C3 on larvae compared to *E. coli*. This may be due to the production of proteases and chitinases, previously found to participate to the larvicidal activity of *S. marcescens* in *Anopheles dirus* (Jupatanakul et al., 2020).

We observed that *S. marcescens* VA is a very efficient colonizer in adult mosquitoes regardless of its ability to synthesize secondary metabolites. This is consistent with previous observations of a very high colonization efficiency by a white strain of *S. marcescens* in *Anopheles* mosquitoes (Wang et al., 2017). Alternatively, a colonization phenotype may appear in a colony with less *Serratia* in its microbiota. Indeed, colonization resistance assays using gnotobiotic mosquitoes (i.e., mosquitoes with a known initial microbiota composition) showed that the initial microbiota impacts the colonization efficiency of other *S. marcescens* strains in *Ae. aegypti* (Kozlova et al., 2020).

Our survival assays using concentrated bacteria were based on two alternative set ups, with or without provision of an additional source of water separately to the sugar solution containing bacteria. We were intrigued to see the extent of which lifespan is prolonged in the presence of water. Median survival was prolonged by 3 days after infection with *S. marcescens* VA. This impact was even stronger in the case of the C3 mutant, where a 12-day extension in median survival was observed. In *Drosophila*, *S. marcescens* oral infection was found to cause thinning of the gut epithelium as a purge mechanism allowing recovery (Lee et al., 2016). We hypothesize that such a defense response may increase the risk of dehydration and/or the need of water for tissue reconstruction. Moreover, mosquitoes may avoid the sugar solution that is contaminated with *S. marcescens* and therefore become dehydrated. Such avoidance has been observed in *Caenorhabditis elegans*, where it is elicited by a serratamolide, serrawettin W2 (Pradel et al., 2007). The observed strong difference between C3 infection in the presence or absence of drinking water may be consistent with such a lack of avoidance, where thirsty mosquitoes would maintain a chronic C3 infection by drinking this contaminated-sugar meal as an alternative to water while C3 infection would be rapidly cleared out in thirsty mosquitoes provided water.

The observed virulence of wt *S. marcescens* VA was reduced in both C1 and C3 when using live bacteria throughout larval development, while the crude extract of C1 had a similar larvicidal activity to wt during late larval development. This difference may be linked with the time of the experiment, as deaths were often observed at least 3 days after the beginning of the experiment during larval development analyses, while impact of the crude extract was only tested over 48 h. Alternatively, it may be explained by differences in the larval stage, where first instar larvae are more sensitive to prodigiosin than third and fourth-instar larvae, as suggested by Suryawanshi et al. (2015) after treating larvae with purified prodigiosin. Indeed, we observed that if larvae do not die during the first instars, they were generally stalled in larval development and able to survive until the end of the 2-week experiment.

Together, our results characterize a new strain of *S. marcescens* bacteria which is virulent in mosquitoes and allowed us to investigate the impact of secondary metabolites produced by this strain on mosquito fitness and development.

## Materials and Methods

### Ethics Statement

Procedures involving animals were carried out in accordance with the French legislation. While experiments described in this manuscript do not directly involve animal experimentation, maintenance of our mosquito colony involves blood feeding of females on anesthetized mice were approved by the Ethics Committee on animal experimentation #069 and carried out under the License 973021. All the procedures were of mild severity and protocols were designed to minimize the numbers of animals used.

### Isolation of *S. marcescens* VA and Generation of Mutants

The wt *S. marcescens* VA strain was isolated from *An. darlingi* mosquitoes reared in the laboratory from field-collected adults. To produce mutant strains, a diluted overnight culture was plated on LB and exposed for 30 s to UV light under a microbiological safety cabinet (Herasafe KS, Thermo Fisher Scientific). Three non-pigmented mutants were selected, C1 to C3. After three passages on LB plates, only mutants C1 and C3 were kept as C2 turned red. Resistance of the wt, C1 and C3 to penicillin-streptomycin (Sigma-Aldrich), gentamycin (Sigma-Aldrich), and kanamycin (Sigma-Aldrich) was tested by depositing an 8 μL droplet of antibiotics at diverse concentrations after plating 1/100 dilution of a *S. marcescens* VA overnight culture on LB-agar (Sigma-Aldrich) and detecting halos in the bacterial lawn after a further overnight incubation at 30°C (Supplementary Table 1). Using this method, we determined that our strains were resistant to a cocktail of 200 U/mL penicillin and 200 μg/mL streptomycin, and we used this condition to select and quantify our *S. marcescens* isolate compared to the rest of bacteria composing the mosquito microbiota in subsequent colonization and infection experiments. An experiment was not considered valid if the non-infected control showed any growth in these conditions.

Growth curves were produced by growing bacteria in liquid LB for 22 h at 30°C and quantifying medium absorbance at 600 nm every 30 min in a FLUOStar Omega plate reader (BMG Labtech).

### Whole-Genome Sequencing of *S. marcescens* VA wt, C1 and C3 Strains

DNA was extracted from overnight cultures using the MagJET genomic DNA kit (Thermo Fisher Scientific) according to the manufacturer’s instructions. Paired-end libraries (150 bp) were prepared using the Nextera XT DNA library preparation kit (Illumina) and sequenced on a MiSeq system by Biofidal (Vaulx-en-Velin, France). Raw reads were demultiplexed and adaptors sequences were trimmed by the sequencing facility. In total, 1.74, 1.63, and 1.73 million reads were obtained for *S. marcescens* VA wt, C1 and C3, respectively. Sequences were quality trimmed using Trimmomatic version 0.39 (Bolger et al., 2014) surviving reads wt: 95.4%, C1: 95.3%, C3: 98.0%) and genomes were assembled *de novo* using SPAdes version 3.14.1 (Nurk et al., 2013). VarScan version 2.3.9 (Koboldt et al., 2012) was used to identify SNPs and indels in C1 and C3 genomes compared to wt.

### Determination of the Inhibition Zone Diameter

*Serratia marcescens* antimicrobial activity was assessed by diffusion in LB-agar. The LB agar plate surface was inoculated by spreading a volume of the test bacteria inoculum over the entire agar surface (OD_600_ = 0.02). Then, 10 μL of overnight culture of *S. marcescens* (OD_600_ = 2) was inoculated as a drop on the same plate. Plates were incubated at 30°C for 24 h. The antimicrobial compounds produced by *S. marcescens* diffuse in the agar medium and inhibit the growth of the microbial strain tested. Then, the diameters of inhibition growth zones were measured using ImageJ^1^.

### Mosquito Colony and Maintenance

*Aedes aegypti* New Orleans mosquitoes were reared in standard insectary conditions, at 28–30°C on a natural 12:12 h light/dark cycle. Larvae were reared on a yeast-based diet, while adults were fed with a sterile 10% (w/v) sucrose solution. Female mosquitoes were blood-fed on anesthetized mice.

### Experimental Mosquito Infection

Bacteria were inoculated from single fresh colonies in LB and incubated at 30°C, shaking at 200 rpm overnight. Bacterial cultures were centrifuged and diluted in a 10% sterile sucrose solution to a final OD_600 nm_ = 1 or 80 (in the latter case, OD_600 nm_ was determined on a 1000-fold diluted sample). After 24 h of starvation (without any sugar solution nor water), five to 7-day-old female mosquitoes were fed with a sterile or bacteria-containing 10% sucrose solution on a sterile cotton ball.

For colonization efficiency assay, six mosquitoes per condition were dissected every 24 h after infection to quantify bacterial contamination.

For survival assays at OD_600 nm_ = 1, bacteria-containing sucrose was replaced every day, while for OD_600 nm_ = 80, a contaminated sucrose solution was provided on day 0 and day 2. Infection was verified 24 and/or 48 h later by culture of six dissected gut homogenates on LB-agar supplemented with 200 U/mL penicillin and 200 μg/mL streptomycin and considered valid if at least 4/6 mosquitoes were infected and if 0/6 non-infected controls (Figures 2B, 4A,B and Supplementary Figures 2, 4) gave rise to any colony or colonies with a clearly distinct morphology (generally no colonies were observed). Two to three biological replicates were performed, based on 150 mosquitoes/condition/replicate.

For microbiota sequencing, 30 mosquitoes/condition were sampled 24 h after infection and mosquitoes fed on a sterile sugar solution were used as a control. Infection was verified by culture of dissected gut homogenates as above.

### Midgut Dissection and CFU Quantification

Female mosquitoes were surface-sterilized in 70% ethanol for 3 min and then rinsed three times in sterile phosphate-buffered saline (PBS). Midguts were dissected, individually transferred in 100 μL sterile LB and homogenized with a sterile plastic grinder. Homogenates were serially diluted and plated on LB agar plates supplemented with 200 U/mL penicillin and 200 μg/mL streptomycin to quantify bacterial loads.

### *16S* rRNA Sequencing

DNA was extracted from pools of 30 midguts using the ZymoBIOMICS MagBead DNA Kit following manufacturer’s instructions. DNA was shipped to Biofidal (Vaulx-en-Velin, France) where it was quantified using the Quantifluor^®^ dsDNA kit (Promega) on a Safire microplate reader (Tecan). The V3–V4 region of the *16S* rRNA gene was amplified using the couple of primers 341F and 805R (Supplementary Table 2), both containing overhang Illumina adapter sequences. PCR reactions consisted of 20 ng of gDNA, 1X HOTBIOAmp^®^ Blend Master Mix with 12.5 mM MgCl_2_ (Biofidal), 1X GC-rich Enhancer (Biofidal), 0.2 mg/mL BSA, 400 nM of each primer. PCR cycle consisted of an initial denaturation step at 96°C for 10 min, 35 cycles of 20 s at 96°C, 30 s at 56°C, 1 min at 72°C and a final elongations step at 72°C for 10 min. PCR products were purified with SPRIselect magnetic beads (Beckman Coulter), quantified using the Quantifluor^®^ dsDNA kit (Promega) on a Qubit^®^ 2.0 fluorometer (Thermo Fisher Scientific) and visualized on a QIAxcel apparatus (QIAGEN). A second PCR (15 cycles) was performed on amplicons to add indexes and P5/P7 adapters. Indexed PCR amplicons were purified, quantified and visualized as described previously. Libraries were pooled after equimolar normalization and sequenced using Illumina MiSeq in paired-end (read length: 300 bp). On average, 34,071 reads were obtained for each sample (median: 35,533, min: 14,558, max: 43,660). After raw reads demultiplexing and adapters trimming from the sequencing facility, qiime2 (Bolyen et al., 2019) was used to inspect read quality. The dada2 plugin (Callahan et al., 2016) was used to trim low-quality portions, denoise reads, remove chimera, and merge FOR and REV reads. Read taxonomy assignation was performed in qiime2 using the SILVA database (Glöckner et al., 2017). Alpha and beta-diversity analyses were performed in qiime2.

### Proteolytic Activity Assay

The culture was incubated for 24 or 48 h, as indicated Figure 3A. It was then centrifuged at 20,000 g for 10 min and the supernatant was filtered through a 0.2 μm pore size membrane. The pellet was washed with PBS and sonicated in a final volume of 300 μL with amplitude 60% for 4 min and then for 30:30 s ON/OFF cycles until the OD reached 0. The suspension was centrifuged for 10 min at max speed to eliminate cell debris. Proteolytic activity was assayed with azocasein as substrate (Sigma-Aldrich) as described previously (Caldas et al., 2002). The sample was mixed 1:1 with a 2% azocasein solution and incubated at 37°C for 1 h in triplicates. Non-digested azocasein was precipitated using 1.3 volumes of 10% TCA (w/v in water) and spun down at 10,000 g for 10 min. The supernatant was then mixed with 2 volumes of 1 M NaOH and absorbance at 440 nm was measured using a microplate reader FLUOstar Omega (BMG Labtech). The blank was obtained by precipitating the sample with the substrate in TCA without any prior incubation. The positive control consisted of 10 mg/mL trypsin (Sigma-Aldrich).

### Hemolytic Activity

#### Blood Agar Plate Test

Bacteria were inoculated from single fresh colonies in LB and incubated at 30°C shaking at 200 rpm overnight. 10 μl of each bacterial culture was spotted on blood agar plates (BD Biosciences) and incubated overnight at 30°C.

### In Liquid Medium

Bacteria were inoculated from single fresh colonies in 3 mL of LB and incubated at room temperature overnight. Human red blood cells (RBCs, Etablissement Français du Sang de Guadeloupe-Guyane) were washed three times with sterile PBS, centrifuged at 1500 g and diluted in PBS to obtain a 1% RBC solution. A total of 200 μL of bacterial cultures was incubated with 200 μL of 1% RBC solution for 24 h at 30°C without shaking. As negative controls, RBCs were incubated with sterile LB and with an overnight *E. coli* HS culture. As positive control, RBCs were incubated with a 0.1% SDS solution. After incubation, RBC suspensions were centrifuged at 12,000 *g* for 5 min and the absorbance of the supernatant at 540 nm was measured. The experiment was performed three times and, for each replicate, three technical replicates were performed.

### Larvicidal Assay in Tubes

Larvicidal assay in tubes was performed as described previously (Falkowski et al., 2016). The crude extract was solubilized in 100% ethanol, adjusted to pH = 6 and a 1.5-fold dilution series of this extract was prepared. This series was further diluted 1% (v/v) in water in 5 mL glass tubes, so that final concentrations ranged from 132 to 1000 ppm. The assay was performed in 20 tubes/concentration, with 5 third or fourth instar larvae in each tube. Larval mortality was recorded 24 and 48 h after exposure. Absolute ethanol was used as a negative control and led to an average 0.75 and 1.8% mortality after 24 and 48 h, respectively.

### Egg Laying

Mosquitoes were fed with a 10% (w/v) sucrose solution contaminated with *S. marcescens* at an OD_600_ = 1 and then with sterile sucrose changed daily. On day 4 post-infection, 10 female mosquitoes were transferred to a cup for blood feeding with bovine blood (kind gift from the Abattoir Régional de Guyane) using a membrane feeding system (Hemotek^®^). Females were kept individually in 30 mL tubes closed with a mesh for individual egg laying, providing sterile sucrose. The number of eggs laid by each female was counted 5 days later.

### Hatching

Individual egg clutches were transferred in water for hatching. For each clutch, 24 larvae were transferred in a 24-well plate to follow larval development and the other larvae were left to develop in a cup for sex ratio assessment.

### Larval Development

Larval development assays were performed as described previously (Romoli and Gendrin, 2020). Eggs were sterilized to obtain axenic larvae. Larvae were transferred individually into 24-well plates (Greiner Bio-One). In each well, 2 mL of bacteria suspension (10^8^ or 10^5^ CFU/mL) and 100 μL of sterile 5% fish food (Tetramin baby) were added. Larval development was followed for 21 days to quantify development success. *E. coli* HS was used as positive control.

### Culture Extraction and HPLC Profiling

The crude extract was obtained as described previously (Patil et al., 2011). Briefly, 2 × 500 mL of culture were incubated for 24 or 48 h at 30 and 37°C (only for WT), shaking at 180 rpm. Cells were harvested by centrifugation at 3900 rpm for 60 min. Pellets were washed four times with 40 mL acidified ethanol (1% v/v HCl 37% in absolute ethanol). The supernatants were dried under low pressure with a rotatory evaporator (Heidolph Laborota 4000) below 40°C.

Prodigiosin hydrochloride (HPLC purity ≥90%, CAS N°: 56144-17-3; Sigma-Aldrich) was dissolved to 0.2 mg/mL in methanol and used without further purification. HPLC samples were prepared from extracts diluted to 10 mg/mL in methanol and 0.22 μm-filtered. For each sample, 10 μL was injected in a Varian 920-LC system equipped with a UV-VIS detector and a photodiode array detector (C_18_ Hypersil Gold column, Thermo Fisher Scientific, 3 μm, 2.1 × 150 mm, flow rate 0.7 mL/min). All samples were analyzed using a linear gradient of H_2_O/CH_3_CN/formic Acid (98:2:0.1 to 2:98:0.1) and detection was performed at 208, 254, 280, and 532 nm.

### NMR Analysis

For NMR analysis, fractions were purified using a hydrophobic C18 reverse phase cartridge, which allows the extraction of non-polar to moderately polar compounds. This purification led to the separation of the C1 extract into three fractions. Only the methanolic fraction (frc3) presents an HPLC profile with compounds showing an absorption maximum at 208 nm, characteristic wavelength of serratamolides (Figures 4A,B and Supplementary Figures 4A,B). Methanolic fraction was dissolved in deuterated methanol (CD_3_OD) prior to introduction into the NMR sample tube. Nuclear magnetic resonance (NMR) spectra (^1^H and 2D sequences) were recorded on a Varian 400 NMR spectrometer equipped with a 5 mm inverse probe (Auto X PGF ^1^H/^15^N-^13^C). ^1^H NMR spectra were recorded at 400 MHz and ^13^C NMR spectra at 100.6 MHz. Chemical shifts are in ppm and coupling constants (J) are in Hz (s for singlet, d for doublet, t for triplet, m for multiplet).

### Gene Complementation in C3

Candidate genes were amplified from wt *S. marcescens* VA using primers indicated Supplementary Table 2 and cloned into pBBR1MCS-2 (kind gift from Kenneth Peterson – Addgene plasmid #85168; Kovach et al., 1995) in *E. coli*. Plasmid sequences were verified by Sanger sequencing. Each recombinant plasmid was then electroporated with a GenePulser (Bio-Rad) in electrocompetent *S. marcescens* C3 cells, prepared with the GenePulser according to the manufacturer’s instructions. For each gene, bacteria were plated on LB supplemented with kanamycin (50 μg/mL) to select those carrying the plasmid.

## Data Availability Statement

The datasets presented in this study can be found in online repositories. The link to the repository and accession number are as follows: https://www.ebi.ac.uk/ena, PRJEB45268.

## Ethics Statement

The animal study was reviewed and approved by the French Direction Générale de la Recherche et de l’Innovation, ethical board # 089, under the agreement # 973021.

## Author Contributions

KH, OR, JS, YEp, EH, YEs, and MG designed the experiments. KH, OR, JS, RA, YEp, VK, EH, and YEs performed the experiments. KH, OR, JS, EH, YEs, and MG analyzed the data. MG drafted the manuscript. KH, OR, and YEs edited the manuscript. All authors accepted the final version of the manuscript.

## Conflict of Interest

The authors declare that the research was conducted in the absence of any commercial or financial relationships that could be construed as a potential conflict of interest.

## Footnotes

1 http://imagej.nih.gov/ij/

## Supplementary Material

Supplementary Figure 1 Growth kinetics of *S. marcescens* VA at 30°C in LB. The average ± SEM of three independent replicates is shown.

Supplementary Figure 2 Chao1 index in mosquitoes contaminated with *S. marcescens* VA. Data were generated from the same experiments as those to produce Figure 1D, and show the average ± SEM of three independent replicates.

Supplementary Figure 3 Quantification of hemolysis activity of *S. marcescens* VA in liquid. *E. coli* and sterile LB (-) were used as a negative control and SDS as a positive control. Data show the average ± SEM of three independent replicates.

Supplementary Figure 4 Peaks detected by HPLC at 208 nm are not found at other wavelengths. HPLC profile of *S. marcescens* crude extracts at 254 nm **(A)** and 280 nm **(B)**. C1-frc3 shows data from the methanolic fraction, which has been analyzed *via* NMR. A. U., arbitrary units.

Supplementary Figure 5 Effect of a low-dose oral infection of *S. marcescens* VA on *Ae. aegypti*. Adult females were fed with a sugar solution contaminated with bacterial strains at OD_600 nm_ = 1. **(A)** Survival analysis. ns, non-significant; ^∗∗∗^*p* < 0.001. **(B)** Number of eggs laid per female and proportion of eggs hatching to larvae after contamination of the mother. **(C)** Proportion of males and females in the progeny at the adult stage. Data show the average ± CI **(A)** and ± SEM **(B,C)** of three independent replicates.

Supplementary Figure 6 Larvicidal activity of a crude extract of *S. marcescens* VA. Lethal concentration of bacterial extracts killing 50% of the larvae (LC_50_) in 48 h. Bacterial extracts were prepared after culturing bacteria at 30 or 37°C for 24 or 48 h. The larvicidal assay was performed on 100 third-to-fourth instar larvae per condition and per replicate. Data show the average of three independent replicates and error bars show SEM.

Supplementary Figure 7 Coloration of *Hfq*-complemented *S. marcescens* C3. Bacteria were grown overnight at 30°C on LB-agar + Kanamycin. *Left*: *Hfq*-complemented C3; *Right*: C3 electroporated with an empty plasmid.
