# Supplementary figures and images for: The Effect of Secondary Metabolites Produced by Serratia marcescens on Aedes aegypti and Its Microbiota
Source: Front Microbiol. 2021 Jul 7;12:645701. doi: 10.3389/fmicb.2021.645701 (PMC8294061; doi:10.3389/fmicb.2021.645701)

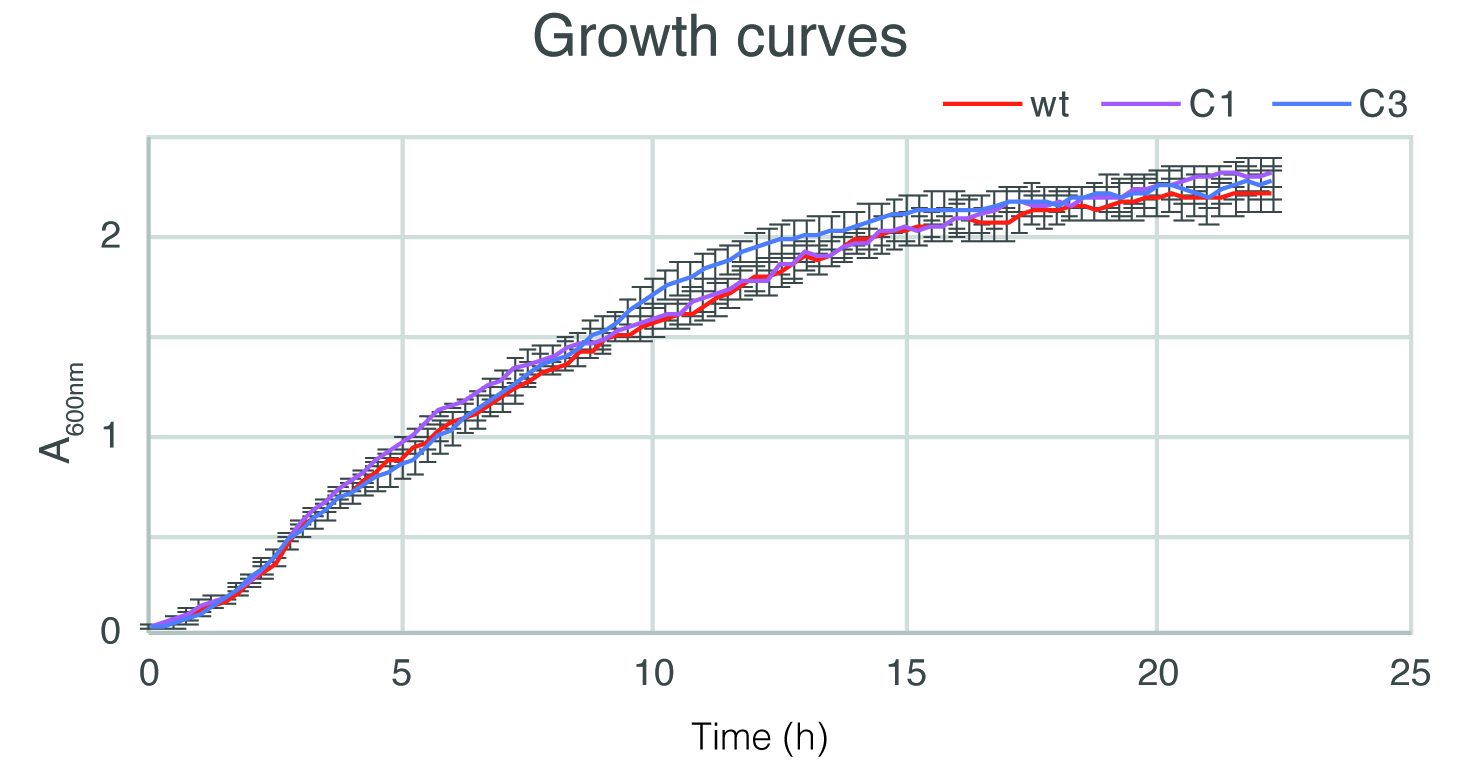

Supplement: Supplementary Figure 1 — Growth kinetics of S. marcescens VA at 30°C in LB. The average ± SEM of three independent replicates is shown. [file Image_1.TIF]

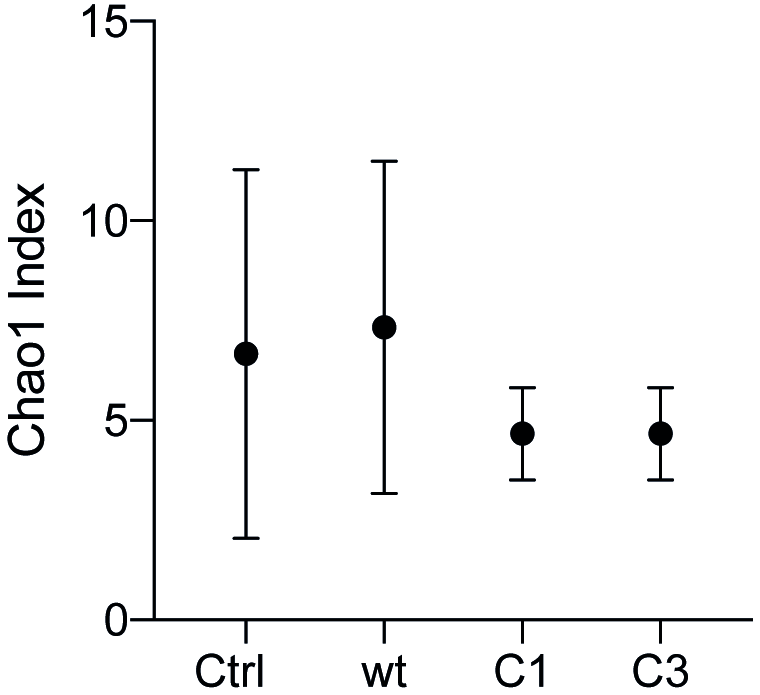

Supplement: Supplementary Figure 2 — Chao1 index in mosquitoes contaminated with S. marcescens VA. Data were generated from the same experiments as those to produce Figure 1D, and show the average ± SEM of three independent replicates. [file Image_2.TIF]

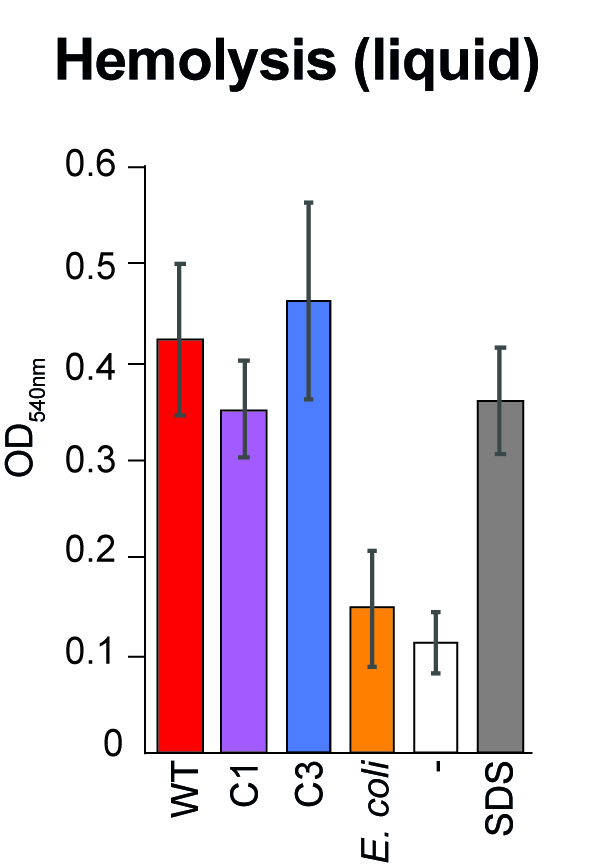

Supplement: Supplementary Figure 3 — Quantification of hemolysis activity of S. marcescens VA in liquid. E. coli and sterile LB (-) were used as a negative control and SDS as a positive control. Data show the average ± SEM of three independent replicates. [file Image_3.TIF]

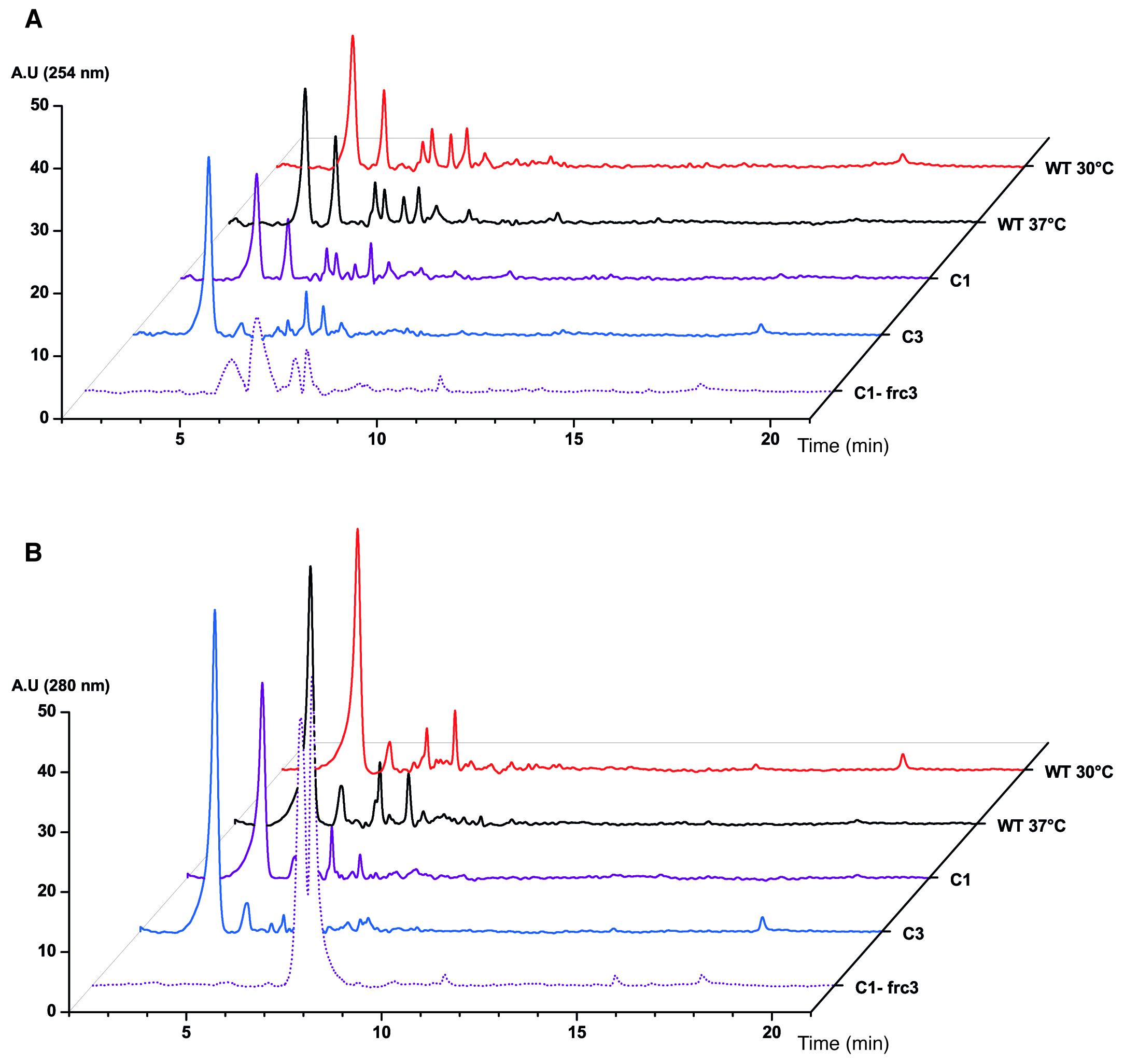

Supplement: Supplementary Figure 4 — Peaks detected by HPLC at 208 nm are not found at other wavelengths. HPLC profile of S. marcescens crude extracts at 254 nm (A) and 280 nm (B). C1-frc3 shows data from the methanolic fraction, which has been analyzed via NMR. A. U., arbitrary units. [file Image_4.TIF]

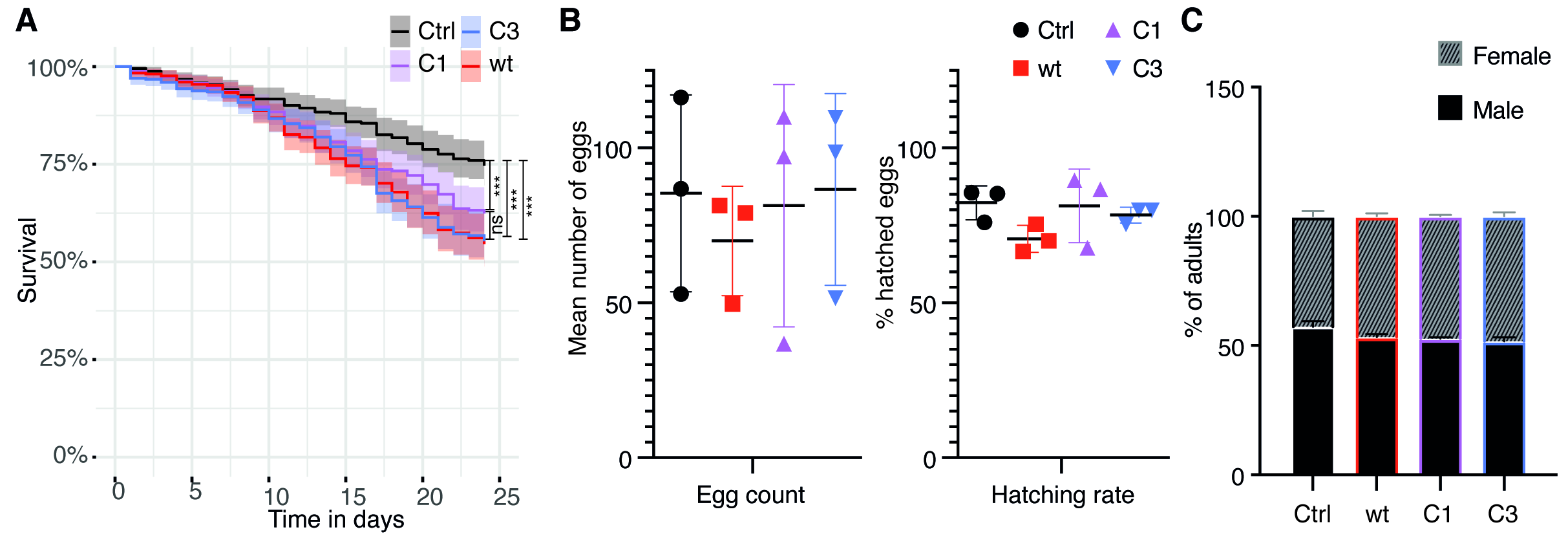

Supplement: Supplementary Figure 5 — Effect of a low-dose oral infection of S. marcescens VA on Ae. aegypti. Adult females were fed with a sugar solution contaminated with bacterial strains at OD600 nm = 1. (A) Survival analysis. ns, non-significant; ∗∗∗p < 0.001. (B) Number of eggs laid per female and proportion of eggs hatching to larvae after contamination of the mother. (C) Proportion of males and females in the progeny at the adult stage. Data show the average ± CI (A) and ± SEM (B,C) of three independent replicates. [file Image_5.TIF]

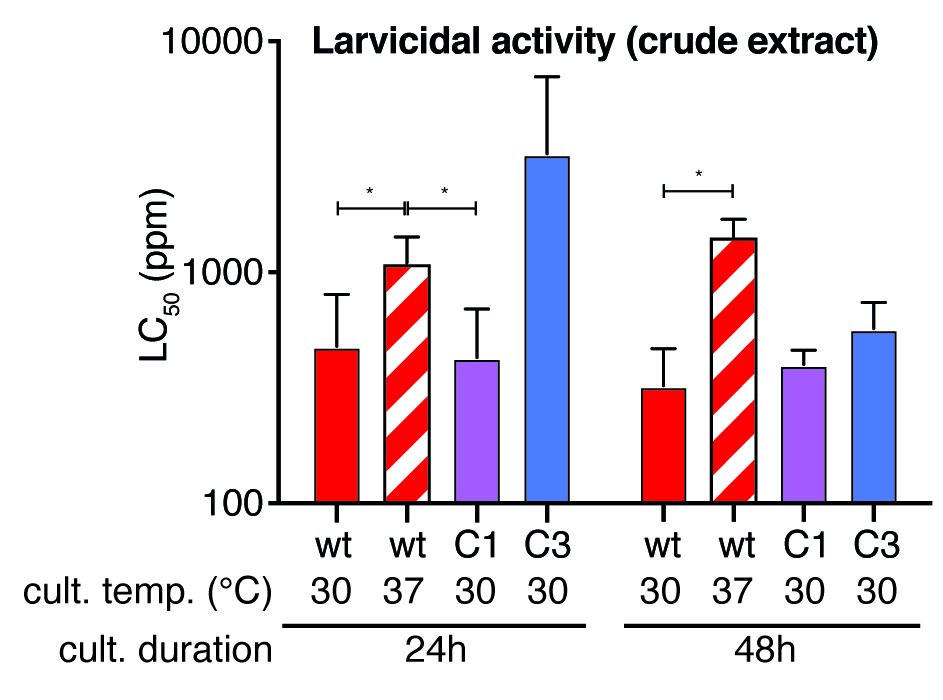

Supplement: Supplementary Figure 6 — Larvicidal activity of a crude extract of S. marcescens VA. Lethal concentration of bacterial extracts killing 50% of the larvae (LC50) in 48 h. Bacterial extracts were prepared after culturing bacteria at 30 or 37°C for 24 or 48 h. The larvicidal assay was performed on 100 third-to-fourth instar larvae per condition and per replicate. Data show the average of three independent replicates and error bars show SEM. [file Image_6.TIF]

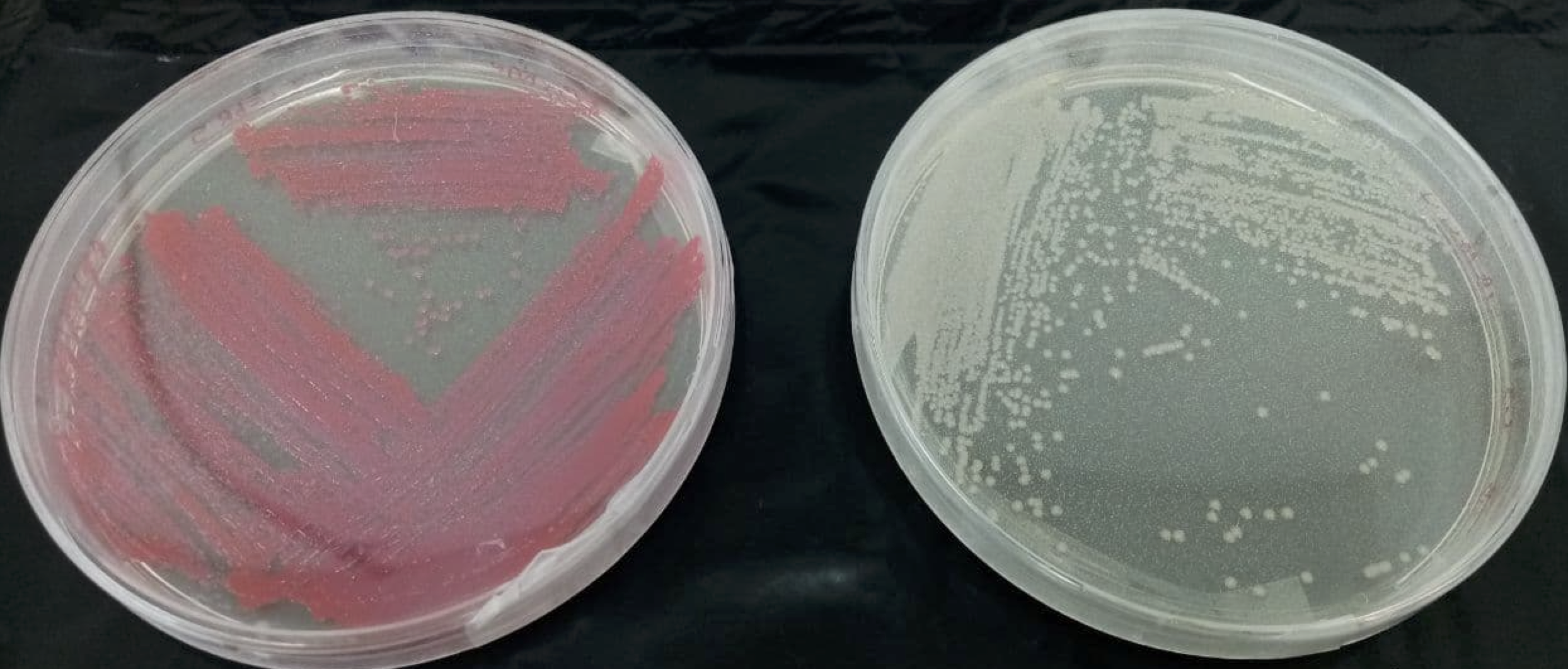

Supplement: Supplementary Figure 7 — Coloration of Hfq-complemented S. marcescens C3. Bacteria were grown overnight at 30°C on LB-agar + Kanamycin. Left: Hfq-complemented C3; Right: C3 electroporated with an empty plasmid. [file Image_7.TIFF]
