# Supplementary material for: The Effect of Secondary Metabolites Produced by Serratia marcescens on Aedes aegypti and Its Microbiota
Source: Front Microbiol. 2021 Jul 7;12:645701. doi: 10.3389/fmicb.2021.645701 (PMC8294061; doi:10.3389/fmicb.2021.645701)
Supplement: Supplementary file 8 [file Table_1.pdf]

Table S1

Antibiotic	Concentration	Halo in the bacterial lawn		
		wt	C1	C3
Penicillin-streptomycin (1x: 100 U/mL penicillin; 100 µg/mL streptomycin)	1x	-	-	-
	2x	-	-	-
	4x	+/-	+/-	+/-
	8x	+	+	+
	16x	+	+	+
Gentamycin (1x: 10µg/mL)	1x	+/-	+/-	+/-
	2x	+	+	+
	4x	+	+	+
	8x	+	+	+
	16x	+	+	+
Kanamycin (1x: 50 µg/mL)	1x	+	+	+
	2x	+	+	+
	4x	+	+	+
	8x	+	+	+
	16x	+	+	+
