# Supplementary material for: The Effect of Secondary Metabolites Produced by Serratia marcescens on Aedes aegypti and Its Microbiota
Source: Front Microbiol. 2021 Jul 7;12:645701. doi: 10.3389/fmicb.2021.645701 (PMC8294061; doi:10.3389/fmicb.2021.645701)
Supplement: Supplementary file 9 [file Table_2.pdf]

Table S2. Primer sequences. For cloning primers, the *Xho*I and *Sac*I restriction sites are indicated in red.

name F	name R	gene name	gene code (ncbi)	sequence F / R	amplicon size (pb)
16S 341F	16S 805R	16S		CCTACGGGNGGCWGCAG / GACTACHVGGGTATCTAATCC	
M13_F	M13_R	plasmid		GTAAAACGACGGCCAG / CAGGAAACAGCTATGAC	
DeoR_*Xho*I_FOR	DeoR_*Sac*I_REV	DeoR/GlpR transcriptional regulator	WP_015377979.1	TTACTCGAGATGATCAATACCGCAAAACG / TTAGAGCTCTTAACGGTCGGCGATAATC	771
NMLTO_*Xho*I_FOR	NMLTO_*Sac*I_REV	N-methyl-L-tryptophan oxidase	WP_015377443.1	TTACTCGAGATGATCGACAGCGCC / TTAGAGCTCTTAGCTGAATCGCTTCAGA	1029
S9_Pept_*Xho*I_FOR	S9_Pept_*Sac*I_REV	S9 family peptidase	WP_015377748.1	TTACTCGAGATGACTTCATTACCCAACAG / TTAGAGCTCTTACGCTGACTTGGGTTT	2094
TolA_*Xho*I_FOR	TolA_*Sac*I_REV	Hypothetical protein - Cell envelope integrity protein TolA	WP_015379201.1	TTACTCGAGATGACGACCGATCAAACC / TTAGAGCTCTTACACCTTTTCCAAACTTTC	2232
RNA_Chap_*Xho*I_FOR	RNA_Chap_*Sac*I_REV	RNA chaperone Hfq	WP_004933678.1	TTACTCGAGATGGCTAAGGGGCAATCTTTG / TTAGAGCTCTTATTCAGCGTCATCGCTTTC	309
ASL_*Xho*I_FOR	ASL_*Sac*I_REV	Argininosuccinate lyase	WP_015379406.1	TTACTCGAGATGGCACTTTGGGGCGG / TTAGAGCTCTCAAGCCAAACGCTGTTTCG	1374
HYP_*Xho*I_FOR	HYP_*Sac*I_REV	Hypothetical protein		TTACTCGAGATGACAGAAAAAACATCTATATCTG / TTAGAGCTCTTATCCTTTGGCGAAGGTT	462
